# Postural Strategies and Sensory Integration: No Turning Point between Childhood and Adolescence

**DOI:** 10.1371/journal.pone.0013078

**Published:** 2010-09-29

**Authors:** Sophie Mallau, Marianne Vaugoyeau, Christine Assaiante

**Affiliations:** Groupe DPA, Pôle 3C - UMR 6149, Université de Provence & CNRS, Marseille, France; Freie Universitaet Berlin, Germany

## Abstract

In this study, we investigated the sensory integration to postural control in children and adolescents from 5 to 15 years of age. We adopted the working hypothesis that considerable body changes occurring during these periods may lead subjects to under-use the information provided by the proprioceptive pathway and over-use other sensory systems such as vision to control their orientation and stabilize their body. It was proposed to determine which maturational differences may exist between the sensory integration used by children and adolescents in order to test the hypothesis that adolescence may constitute a specific phase in the development of postural control. This hypothesis was tested by applying an original protocol of slow oscillations below the detection threshold of the vestibular canal system, which mainly serves to mediate proprioceptive information, to the platform on which the subjects were standing. We highlighted the process of acquiring an accurate sensory and anatomical reference frame for functional movement. We asked children and adolescents to maintain a vertical stance while slow sinusoidal oscillations in the frontal plane were applied to the support at 0.01 Hz (below the detection threshold of the semicircular canal system) and at 0.06 Hz (above the detection threshold of the semicircular canal system) with their eyes either open or closed. This developmental study provided evidence that there are mild differences in the quality of sensory integration relative to postural control in children and adolescents. The results reported here confirmed the predominance of vision and the gradual mastery of somatosensory integration in postural control during a large period of ontogenesis including childhood and adolescence. The youngest as well as the oldest subjects adopted similar qualitative damping and segmental stabilization strategies that gradually improved with age without reaching an adult's level. Lastly, sensory reweighting for postural strategies as assessed by very slow support oscillations presents a linear development without any qualitative turning point between childhood and adolescence.

## Introduction

It is well known that control of posture is a complex multisensorial task based on vestibular, visual and somatosensory information - arising from sensory sources such as muscle, skin, and joints. Each sensory pathway has a specific activation threshold and sensitivity. However, there does not appear to be sensory hierarchy to maintain postural orientation and stabilization. The selection and physiological reweighting are made according to the context and the developmental period of each subject. Both children and adults make use of visual, vestibular and proprioceptive information to control their body posture, but the respective contributions of these inputs vary during ontogenesis [1].

Several studies have shown that postural oscillations decrease with age from childhood to adulthood [2]–[6] suggesting that children control their posture less efficiently than adults. Studies on the development of balance control have reported the existence of marked differences with respect to adults, especially in terms of the segmental stabilization occurring at head, trunk and pelvis levels [1], [7]–[8]. According to their ontogenetic model of balance control, Assaiante and Amblard [1] assumed that the various balance strategies adopted by children as well as by adults involve to take into account two main functional principles of spatial organization. The first concerns the choice of the stable reference frame on which the equilibrium control is based, and the second concerns the gradual mastery of the degrees of freedom of the various body joints. The choice of the stabilized anatomical segment of reference as well as the character of coupling between articulations depends on the dynamic constraints determining the difficulty of a motor task, the environment and the characteristics of each developmental period. For example, the pelvis constitutes the first stable reference frame, around which balance control can be built up, as soon as independent locomotion is acquired [8]. By contrast, controlling head stabilization during locomotor activities constitutes a complex motor skill that takes a long time to mature during childhood [7].

Classically in the literature one of the earliest studies reported was on visual contribution to postural control [9], primarily because vision is easy to manipulate and by the fact that when the eyes are closed, stability decreases. Spontaneous oscillations of the body cause slip of the image of the retina that is subsequently used to stabilize the body. Thus detection of visual movement allows body stabilization. This coupling between visual perception and action has been reported efficient in newborn babies to generate postural activity at the neck level in response to the visual flow produced by an appropriate moving room [10]. In children, it is well established that visual cues play a prominent role in balance control in postural and locomotor tasks Shumway-Cook and Woollacott, 1985 [11]–[14]. In a recent study, Ferber-Viart and colleagues [15] using Equitest computerized dynamic posturography in children from 6 to 14 years showed that children had lower equilibrium scores than young adults, especially when visual information was not available or was incorrect. According to these authors, this predominant visual involvement in balance control in children needs to be investigated further since it has been shown that ocular disorders were often responsible for balance abnormalities during childhood [16]. Paradoxically, careful examination of eye movement development in relation to balance control during childhood and adolescence has not been carried until now. Some earlier studies in adults only focussed on saccades recording indicate improvement of postural stability with eyes [17]–[19], while more recent studies [20], [21] suggest the opposite.

From the three sensory systems governing postural control, proprioceptive inputs are thought to have the greatest influence in the detection of body sway [22]. Indeed, many developmental studies [5], [23], [24] reported the importance of the proprioceptive system for postural control in children. For example, Hirabayashi and Iwasaki [25] reported that the function of the somatosensory system developed early and reached the adult level by the age of 3 or 4 years. Nevertheless, from tendinous vibration studies in children from 7 to 15 years of age, Perterka and Black [4] as well as Hytonen et al. [5] reported that children show a delay in the maturation of the integration of the proprioceptive cues to improve postural control. Moreover, a recent study reported that healthy 7 to 12-year-old-children were unable to use somatosensory inputs in order to limit the body sway generated by dynamic visual cues to the same extend as adults, suggesting that the sensory integration of the somatosensory cues is still developing at 12 years of age [26].

Recently Bair and colleagues [27] hypothesize, as did Forssberg and Nashner [28], that improvements in postural control with development may be due in part to better ability in sensory reweighting. Mature sensory reweighting uses information from all sensory modalities simultaneously, reflecting the fact that a change in one sensory input leads to change in response to all sensory inputs. Bair and colleagues [27] have recently emphasized the link between adaptive reweighting mechanism and an anticipatory process requiring a sophisticated internal model that can predict the sensory consequences of self motion. Taking into account all these developmental results on sensory integration, the control of posture in humans is very complex and involves virtually all parts of the nervous system. Neuroscience research has made important contributions to our understanding of development by demonstrating that the brain is far more plastic at all ages than previously thought and the remarkable role of experience in shaping the mind, brain and body [29]. Therefore, it is not too surprising that the development of postural control is a long-term process, which is not complete at preschool age, but lasts till adolescence.

In the current study, we applyed an original protocol of slow oscillations of the platform on which the subjects were standing at the frequency below the detection threshold of the vestibular canal system, in order to observe adjustments that are primarily driven by the proprioceptive system, especially when the eyes are closed. We adopted the working hypothesis that considerable body changes occurring during these periods may lead subjects to under-use the information provided by the proprioceptive pathway and over-use other sensory systems such as vision to control their orientation and stabilize their body. Lastly, it was proposed to determine which maturational differences may exist between the sensory integration used by children and adolescents in order to test the hypothesis that adolescence may constitute a specific phase in the development of postural control.

## Materials and Methods

### 1. Subjects

This experiment was approved by the local ethical committee i.e CPP Sud-Méditerranée I, therefore has been performed in accordance with the ethical standards of the Declaration of Helsinki. All the subjects and their parents gave their written (verbal for the youngest children) informed consent prior to the study.

55 subjects participated in this study: 35 healthy children from 5 to 13 years and 20 adolescents from 14 to 15 years. Four groups were compared: a group of 10 children aged from 5 to 6 years (mean 5 years 7 months, SD+/−6 months; 5 girls and 5 boys), a group of 13 children aged from 7 to 10 years (mean 8 years 6 months, SD+/−15 months; 7 girls and 6 boys), a group of 12 children aged from 11 to 13 years (mean 12 years 2 months, SD+/−12 months; 6 girls and 6 boys), and a group of 20 adolescents aged from 14 to 15 years (mean 14 years 9 months, SD+/−8 months; 10 girls and 10 boys).

The children were all primary school pupils and the adolescents were all high school students. All subjects presented normal motor function and performed sports activities in their everyday life, without any motor disorders suspected. The age, height, weight and sex of each children and adolescent participant are reported in Table 1.

**Table 1:** Age of the subjects.

Group	5–6	7–10	11–13	14–15
**Number of subjetcs**	10	13	12	20
**Boys**	5	6	6	10
**Girls**	5	7	6	10
**Age**	5 years 7 months +/−6 months	8 years 6 months +/−15 months	12 years 2 months +/−12 months	14 years 9 months +/−8 months
**Mean Height (cm), (SD)**	115 (+/−3)	132 (+/−8)	151 (+/−8)	168 (+/−9)
**Mean Weight (kg),(SD)**	20.2 (+/−2.39)	26.7 (+/−7.36)	41.7 (+/−8)	58.5 (+/−12.32)

### 2. Experimental set-up

Subjects stood on a motorised uni-directional rotating platform with their eyes open (EO) or closed (EC). The platform was rotated sinusoidally at 0.01 Hz and at 0.06 Hz (10 degrees peak to peak) in the roll direction. They had to maintain a vertical posture as steadily as possible, keeping their feet 15 cm apart without flexing their knees. A similar experimental set-up was successfully used in young adults [30] as well as in adults with Parkinson's disease [31] and cervical dystonia [32].

At the lowest frequency, the maximum angular accelerations of the platform were thus well below the vestibular canal's perception threshold, namely 0.2°/s^2^ [33]. Therefore, at this frequency, if any angular head accelerations occurred beyond this threshold value, they would not result directly from the platform movements and would not be involved in correcting the experimentally induced postural disturbances. In other words, postural adjustments in this case, would be mainly related to visual and proprioceptive feedback. Moreover, in the condition where the subjects were tested with their eyes closed at a frequency of 0.01 Hz, the subjects' use of proprioceptive cues was mainly tested. By contrast, at the highest frequency (0.06 Hz), the slow sinusoidal oscillations in the frontal plane applied to the supporting platform were above the detection threshold of the semicircular canal system that implied that vestibular cues were also available with proprioception, and with or without vision to control posture. Even at the lowest frequency, all the subjects (children and adolescents) were aware that the platform was rotating.

**Figure 1:**
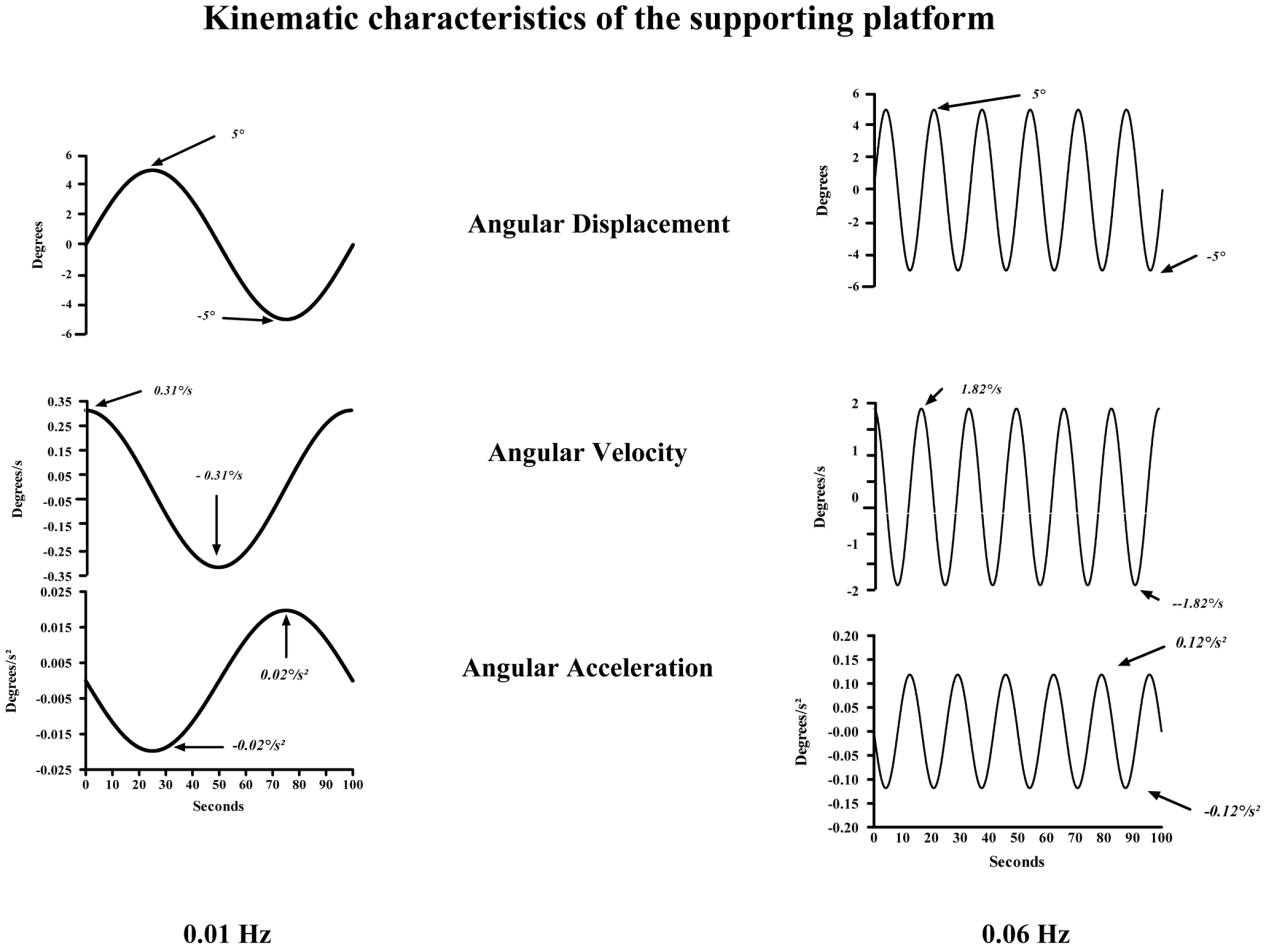
Characteristics of the supporting platform's movement at 0.01 Hz (left panels) and at 0.06 Hz (right panels). The first curves represent the angular displacements of the supporting platform. The seconds represent the angular velocity of the supporting platform and the thirds represent the angular acceleration of the supporting platform. The arrows indicate the peaks of inclination, of velocity and of acceleration.

The trials' duration was 106 seconds, including a complete cycle of angular platform movement at the lower frequency and 6 at the higher frequency. The figure 1 represents the characteristics of the support's movements.

### 3. Data collection and kinematic analysis

Data collection was performed with the SMART automatic motion analyser (eMotion) working at 120 Hz, using passive body markers.

**Figure 2:**
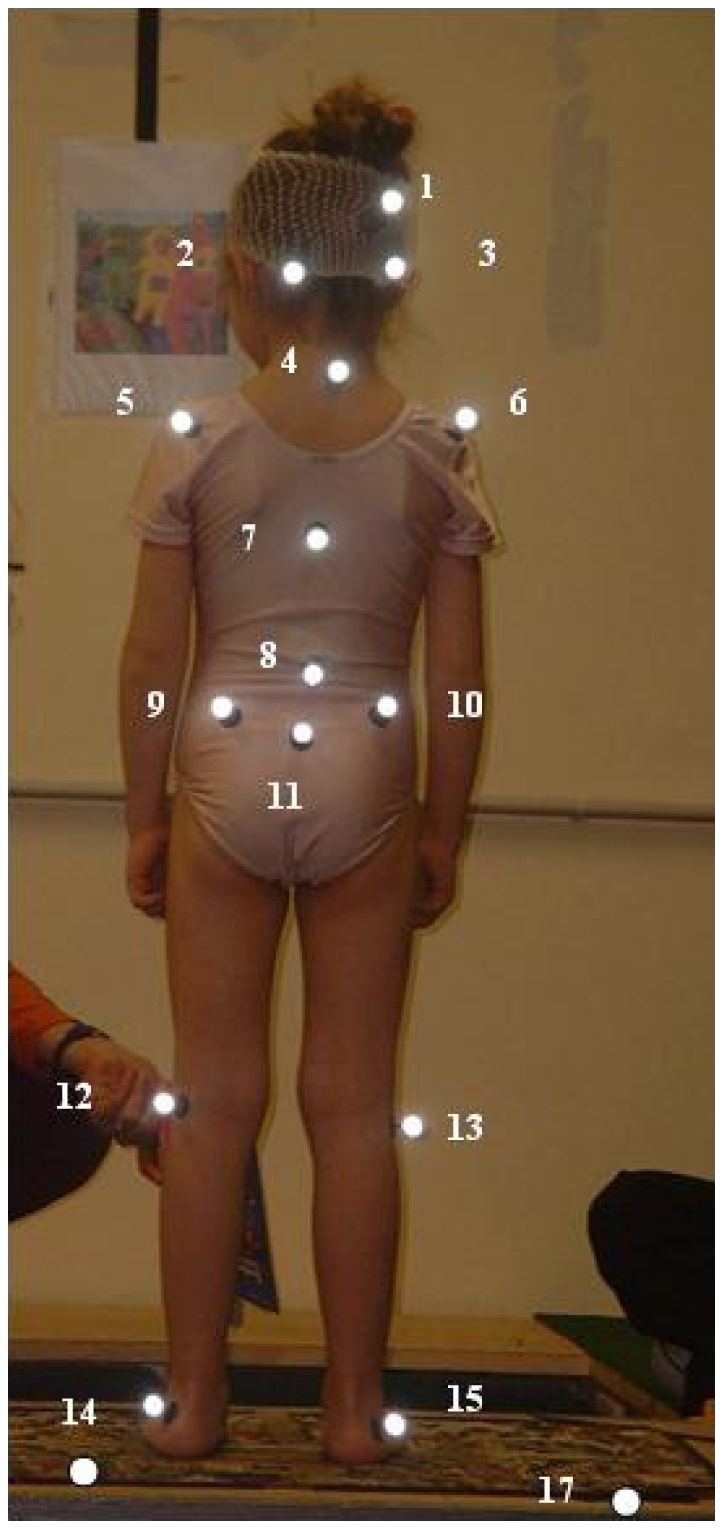
Subject's photography representing the markers' position. The 15 markers were placed symmetrically in pairs on the subject's back at the following sites: top of the head (1), mastoid (2, 3), acromion process (5, 6), spinal process of C7 (4), L2 (7) and T6 (8), on the sacrum (11), posterior-superior iliac crest (9, 10), lateral tibial plate (12, 13), external malleolus (14, 15). Two last markers were also placed on the platform (16, 17) to measure its lateral movements.

Subjects performed the task facing six SMART TV cameras and wearing 15 markers (15 mm in diameter) onto the skin, placed symmetrically on the child's back as indicated in figure 2. Two supplementary markers were placed on the supporting surface in order to record its movements.

### 4. Controlled variables

Four trials were run with each subject investigated in each experimental condition. The trials were proposed in a pseudo-randomized order. Three controlled variables were used to estimate both segmental orientation (sequential orientation, angular dispersions) and stabilization (anchoring index). The variables were averaged in all subjects and all trials in each experimental condition.

#### a. Sequential orientation

Sequential orientation values were calculated on each body segment within each tenth of a cycle (corresponding to 10 s at 0.01 Hz and to 1.67 s at 0.06 Hz) of platform movement, in order to assess the time course of the segmental orientation process, especially at the maximum platform tilt angle, i.e. under maximum postural perturbation conditions.

#### b. Angular dispersions

During each trial the absolute (with respect to external axes) head, shoulder, trunk and pelvis roll angles were computed. The oscillations induced in each anatomical segment by the movement of the supporting platform were assessed in terms of segmental angular dispersion: at each trial, the standard deviation (i.e the dispersion) of over all angle orientation values during the considered trial was calculated.

The angular dispersion gives a first indication of the oscillations of a given segment during the perturbation. This variable indirectly provides some information about the attenuation of the perturbation at the anatomical level considered. When the angular dispersion of a body segment is smaller than that of another body segment, this indicates that the first body segment has moved less than the second one, or in other words, that the perturbation is more attenuated at the former anatomical level.

#### c. Anchoring Index

**Figure 3:**
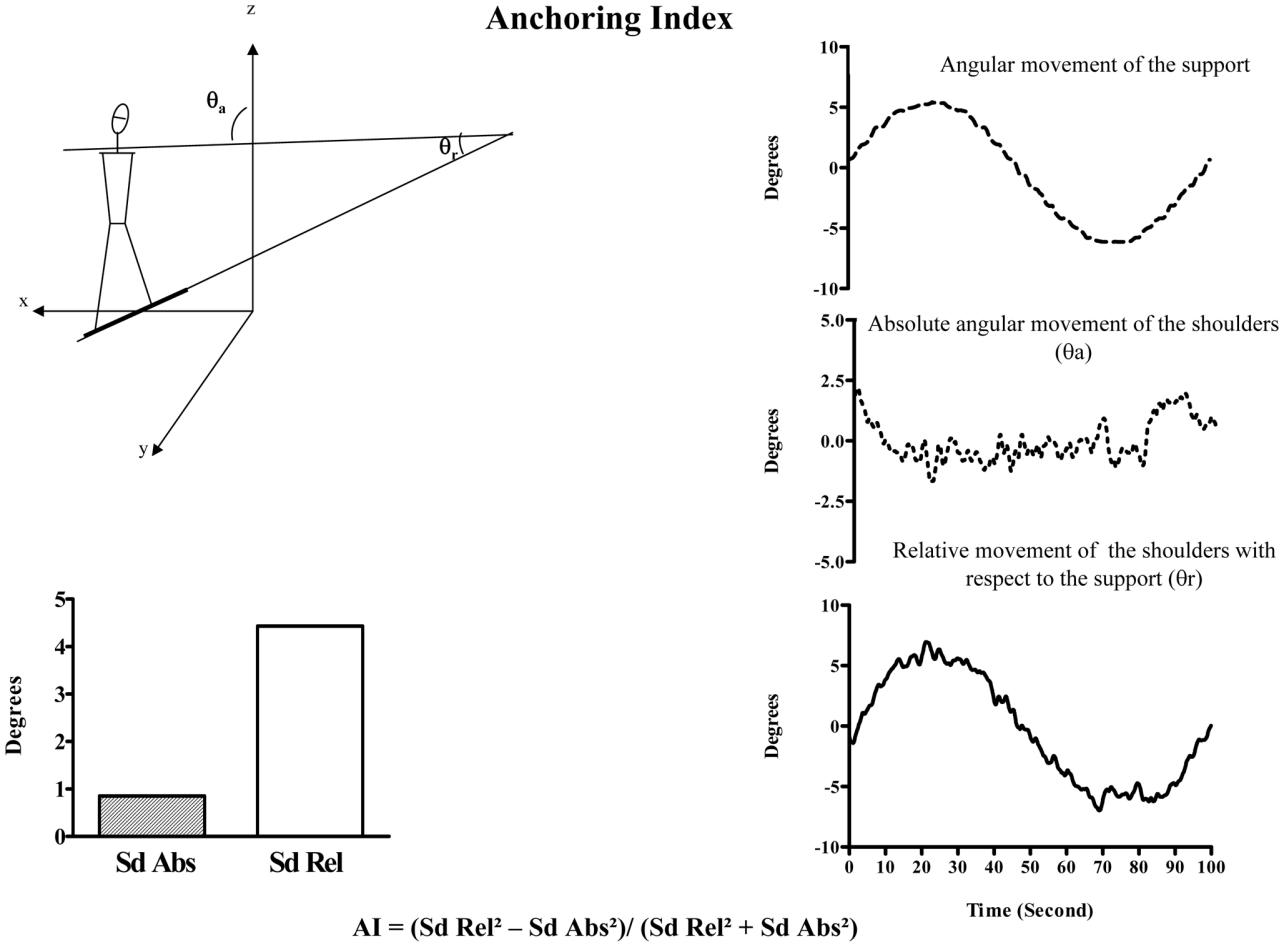
Left upper panel: Diagram of the shoulder roll angle with respect to the external axis, θ_a_, and with respect to the supporting platform, θ_r_. With x: lateral axis, y sagittal axis and z vertical axis. Right panel: angular roll displacement of the supporting platform (upper trace), the absolute angular displacement of the shoulders (middle trace) and the relative angular movement of the shoulders with respect to the supporting calculated every 8.33 ms during a trial using the formula: 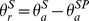 with 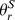, the angular orientation of the shoulders relative to the support, and 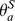 and 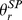 are the absolute shoulders and support angular orientations,s respectively. (Lower trace). Left lower panel: Diagram of the absolute (Sd Abs) and relative (Sd Rel) roll dispersions of the shoulders, according to the definition of the anchoring index (AI). Formula of the AI = (Sd Rel^2^−Sd Abs^2^)/(Sd Rel^2^+Sd Abs^2^) where Sd Abs is the standard deviation of the angular distribution about the roll of the segment under investigation with respect to the absolute allocentric reference (absolute vertical direction) value and Sd Rel is the corresponding standard deviation of the angular distribution with respect to the moving platform. In this example, AI is positive, which means that the shoulders are stabilized in space independently of platform movements.

Segmental stabilization was defined in terms of the global anchoring index calculated during the whole cycle of perturbation [7], [30], [31], [34]–[37]. The segmental anchoring index was used to compare the stabilization of a given segment with respect to both an external reference value and the moving platform. AI was calculated for each trial as follows, as shown in figure 3. A positive segmental value indicates a better segmental stabilization along the absolute vertical axis than in response to the moving platform, whereas a negative value indicates a better segmental stabilization on the platform than on the external absolute axis. The segmental anchoring index was therefore used to compare the level of stabilization of a given segment achieved by the subject in relation to the gravity vertical or to the biased orientation of the supporting platform.

### 5. Statistical analysis

Descriptive statistics are reported as median and interquartiles. The developmental effect was analyzed with an ANOVA of Kruskal-Wallis by ranks. In the case of a global significant age effect, in order to isolate the group or groups that differ from the other, we have realized multiple comparison procedures using the Dunn's Method. The frequencies and vision effects were tested using Wilcoxon's signed rank test for within-subject comparisons. Since the anchoring index is in the −1 to +1 range, we used a z transform to convert the values into an unbiased Gaussian distribution. Differences with a p value <0.05 were considered to be statistically significant.

## Results

### 1. Sequential Orientation

**Figure 4:**
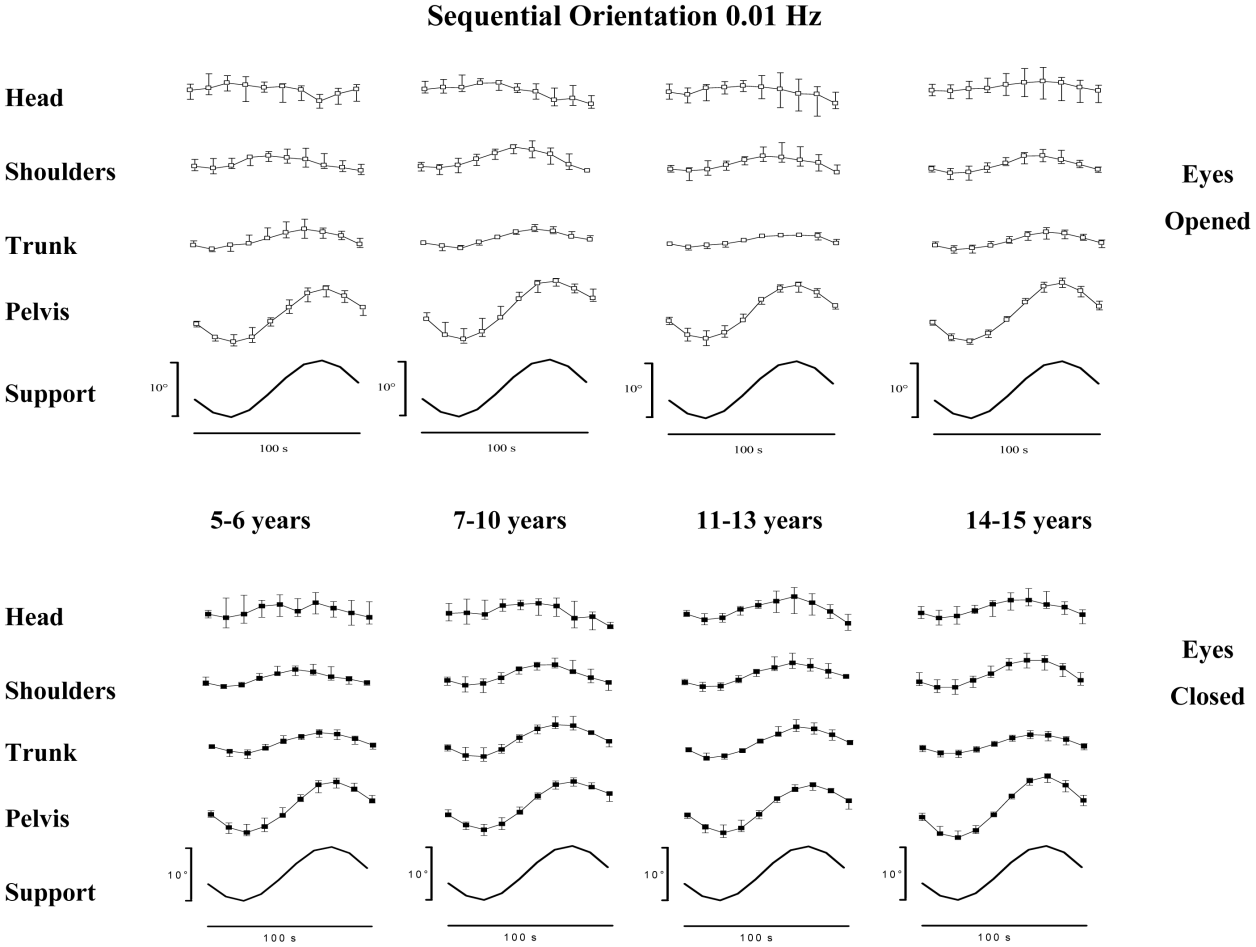
Median and quartiles of sequential orientation (degrees) of head, shoulders, trunk, pelvis and support (top to down) with eyes open (top panel) and eyes closed (down panel) in subjects from 5 to 15 years at 0.01 Hz.

**Figure 5:**
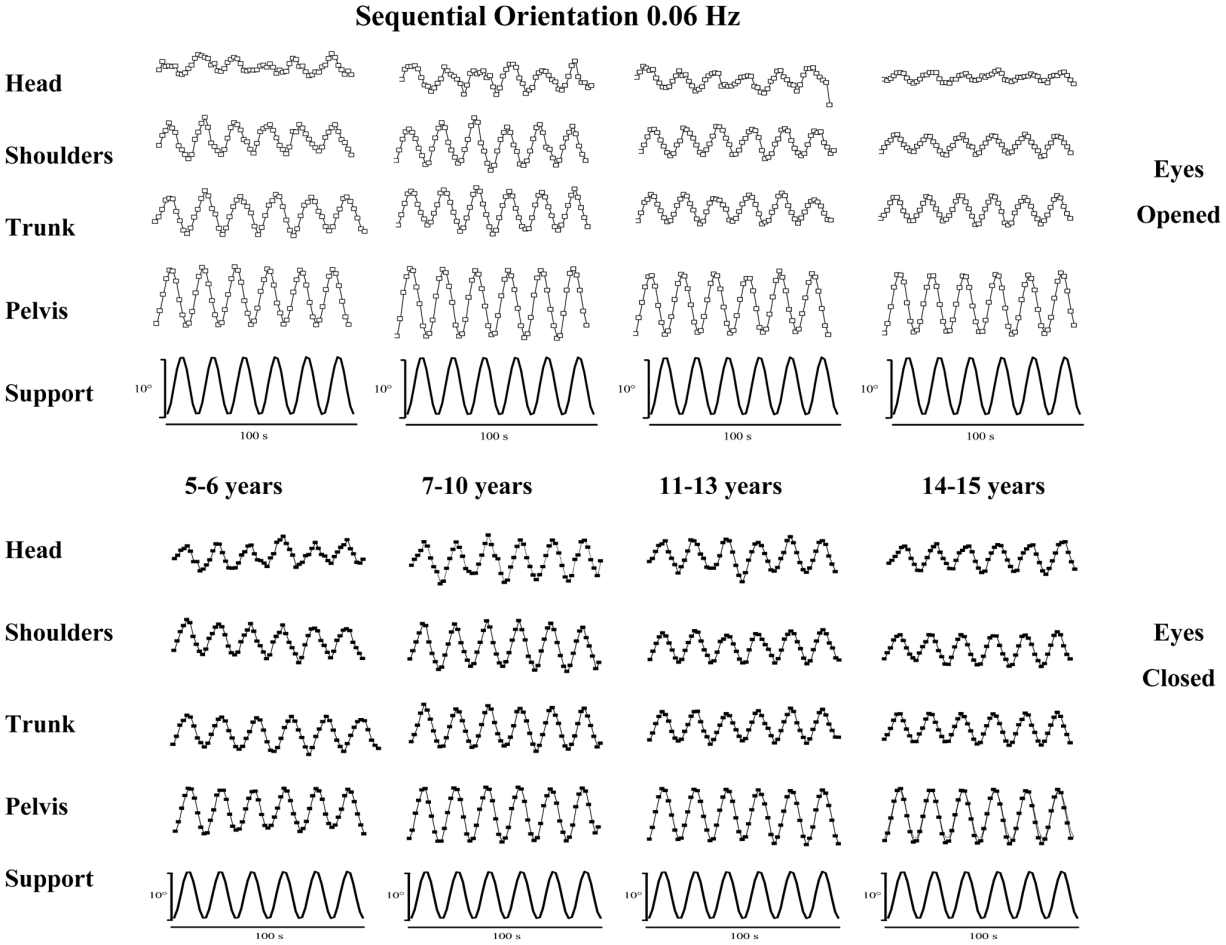
Median and quartiles of sequential orientation (degrees) of head, shoulders, trunk, pelvis and support (top to down) with eyes open (top panel) and eyes closed (down panel) in subjects from 5 to 15 years at 0.06 Hz.

The sequential orientations of each segment considered, with vision (top of the figure) and without vision (bottom of the figure) for each group of subject are shown at 0.01 Hz in figure 4 and at 0.06 Hz in figure 5.

In all groups of subjects, whatever the frequency and the visual condition the pelvis followed the movements of the supporting platform. By contrast, a gradual attenuation of the oscillations occurred in the higher anatomical segments. This damping was more important at the head's level and improved with vision, in particular at the higher frequency. The attenuation of the oscillations induced in the anatomical segments was assessed in terms of the segmental angular dispersions.

### 2. Angular dispersions

**Figure 6:**
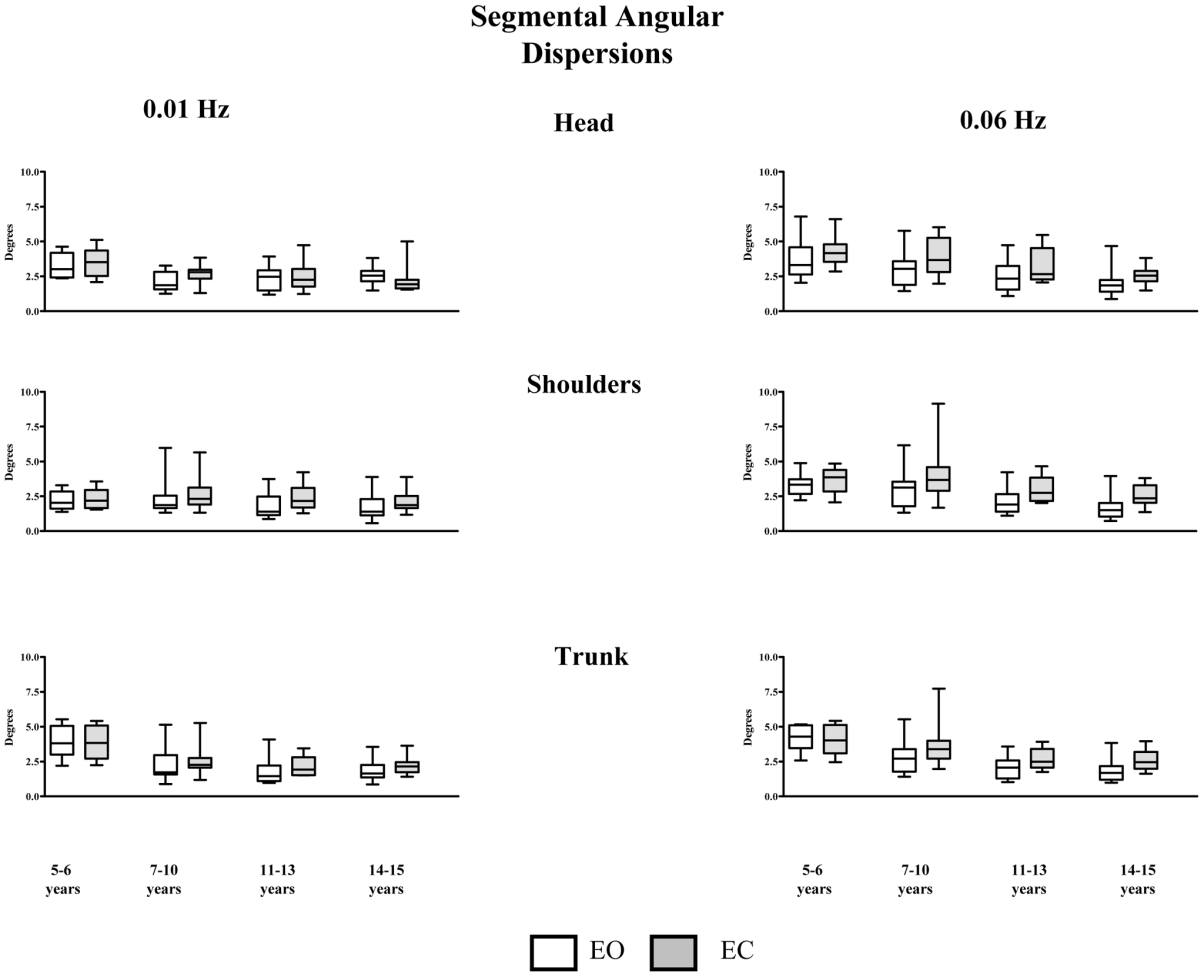
Box and whisker -median and interquartiles range maximum and minimum- of roll head, shoulders, trunk and pelvis (top to down) angular dispersion (degrees), with eyes open (white) and eyes closed (grey) in subjects from 5 to 15 years at 0.01 Hz (left panel) and at 0.06 Hz (right panel).

The angular dispersions of each segment considered, with and without vision, at the lower frequency 0.01 Hz (left part) and at the higher frequency 0.06 Hz (right part) from 5 to 15 years of age, are shown in figure 6.

#### a. Age effect

***At the head level***, at 0.01 Hz, with and without vision, the Kruskall Wallis analysis revealed a significant age effect (EO, H (3, 51) = 13.44, p<.01, EC H (3, 53) = 13.35, p<.01). The head angular dispersion decreased significantly as a function of age. More precisely, the multiple comparison procedure revealed that head angular dispersion of the youngest subjects was significantly lower as compared with those of the oldest subjects (Q = 2.89, p<0.005 and Q = 3.56, p<0.05 for EO and EC respectively).

***At the shoulders level***, at 0.01Hz the age effect was significant only with vision (H (3, 54) = 8.55, p<.05). Nevertheless, the decrease of the angular dispersions of the shoulders from the oldest subjects to the youngest was so moderate that the multiple comparison procedure pairwise did not allow isolating the group that differ from the others. At 0.06 Hz, with vision as well as without vision, the angular dispersion of the shoulders decreased significantly from the youngest subjects to the oldest (H (3,54) = 19.38 and H (3,54) = 14,131 for EO and EC conditions respectively). More precisely, the pairwise multiple comparison procedure revealed that the groups of 5–6 years old (yo) and the group of 7–10 yo presented significant higher shoulders' angular dispersion as compared with those of the 14–15 yo (Q = 3.94 and 3.09, p<0.005 for 5–6 yo and 7–10 yo respectively).

***At the trunk level***, at 0.01 Hz, whatever the visual condition, the Kruskal Wallis analysis did not reveal any age effect. At 0.06 Hz, with vision, the trunk angular dispersion decreased significantly from the youngest subjects to the oldest (H (3,54) = 17,259). More precisely, the pairwise multiple comparison procedure revealed that the group of 5–6 yo and the group of 7–10 yo presented significant higher trunks' angular dispersions as compared with those of the 14–15 yo (Q = 3.64 and 12.09, p<0.005 for 5–6 yo and 7–10 yo respectively). Without vision, the age effect was also significant (H (3, 54) = 9.18, p<.05). Nevertheless, the decrease of the angular dispersions of the trunk from the youngest subjects to the oldest was so moderate that the multiple comparison procedure did not allow isolating the group that differ from the others.

***At the pelvis*** level, whatever the visual and the frequency condition, no age effect was reported. Pelvis angular dispersions displayed little variations around 4 degrees for all groups of subjects.

#### b. Visual effect

In order to assess the effects of vision on each group of subjects, the segmental angular dispersions measured with vision were compared with those measured without vision using a Wilcoxon analysis. The statistical values of this analysis are given on table 2 for 0.01 Hz and 0.06 Hz.

**Table 2:** Results of the Wilcoxon analysis testing the visual effect in angular dispersion.

		0,01				0,06			
		5–6	7–10	11–13	14–15	5–6	7–10	11–13	14–15
**Head**	W	NS	54	NS	122	NS	NS	68	178
	p	P>0,05	P<0,05	P>0,05	P<0,05	P>0,05	P>0,05	P<0,01	P<0,001
**Trunk**	W	NS	NS	46	178	NS	66	60	200
	p	P>0,05	P>0,05	P<0,05	P<0,001	P>0,05	P<0,001	P<0,01	P<0,001
**Shoulders**	W	NS	−66	−78	−210	NS	−46	−62	−169
	p	P>0,05	P<0,01	P<0,001	P<0,001	P>0,05	P<0,05	P<0,05	P<0,001
**Pelvis**	W	NS	NS	NS	NS	NS	NS	NS	NS
	p	P>0,05	P>0,05	P>0,05	P>0,05	P>0,05	P>0,05	P>0,05	P>0,05

NS: Non significant.

The statistical analysis revealed significant increases in angular dispersion with EC as compared to EO as follows: at 0.01 Hz, at the head shoulders and trunk for the oldest group 14–15 yo; at the shoulders and trunk for 11–13 yo and at the head for 7–10 yo; at 0.06Hz at the head shoulders and trunk for 11–15 yo, and at the shoulders and trunk for 7–10 yo. No significant difference was found at the pelvis for any group, and in the 5–6 yo whatever the anatomical level no significant.

#### c. Frequency effect

The statistical values of this analysis are given on table 3 for 0.01 Hz and 0.06 Hz.

**Table 3:** Results of the Wilcoxon analysis testing the frequency effect in angular dispersion.

		0,01				0,06			
		5–6	7–10	11–13	14–15	5–6	7–10	11–13	14–15
**Head**	W	NS	−52	−46	−127	−49	−62	−66	−200
	p	P>0,05	P<0,05	P<0,05	P<0,01	P<0,05	P<0,01	P<0,001	P<0,001
**Trunk**	W	NS	−50	−60	−161	−51	NS	−52	−210
	p	P>0,05	P<0,05	P<0,01	P<0,001	P<0,01	P>0,05	P<0,05	P<0,001
**Shoulders**	W	NS	−66	−78	−210	NS	−46	−62	−169
	p	P>0,05	P<0,01	P<0,001	P<0,001	P>0,05	P<0,05	P<0,05	P<0,001
**Pelvis**	W	NS	NS	NS	NS	NS	NS	NS	NS
	p	P>0,05	P>0,05	P>0,05	P>0,05	P>0,05	P>0,05	P>0,05	P>0,05

NS: Non significant.

The statistical analysis revealed significant increases in angular dispersion at 0.01 Hz as compared to 0.06 Hz as follows: with vision, at the head, shoulder and trunk level for the 5–6 yo, at the head and shoulders levels for the 7–10 yo, at the trunk level for the 11–13 yo; without vision at the head, shoulders and trunk level for the 5–13 yo, at the head and shoulders levels for the 14–15 yo. At the head level head, with vision the statistical analysis revealed a significant decrease of the angular dispersion for the 14–15 yo. No significant difference was found at the pelvis for any group,

### 3. Anchoring index (AI)

**Figure 7:**
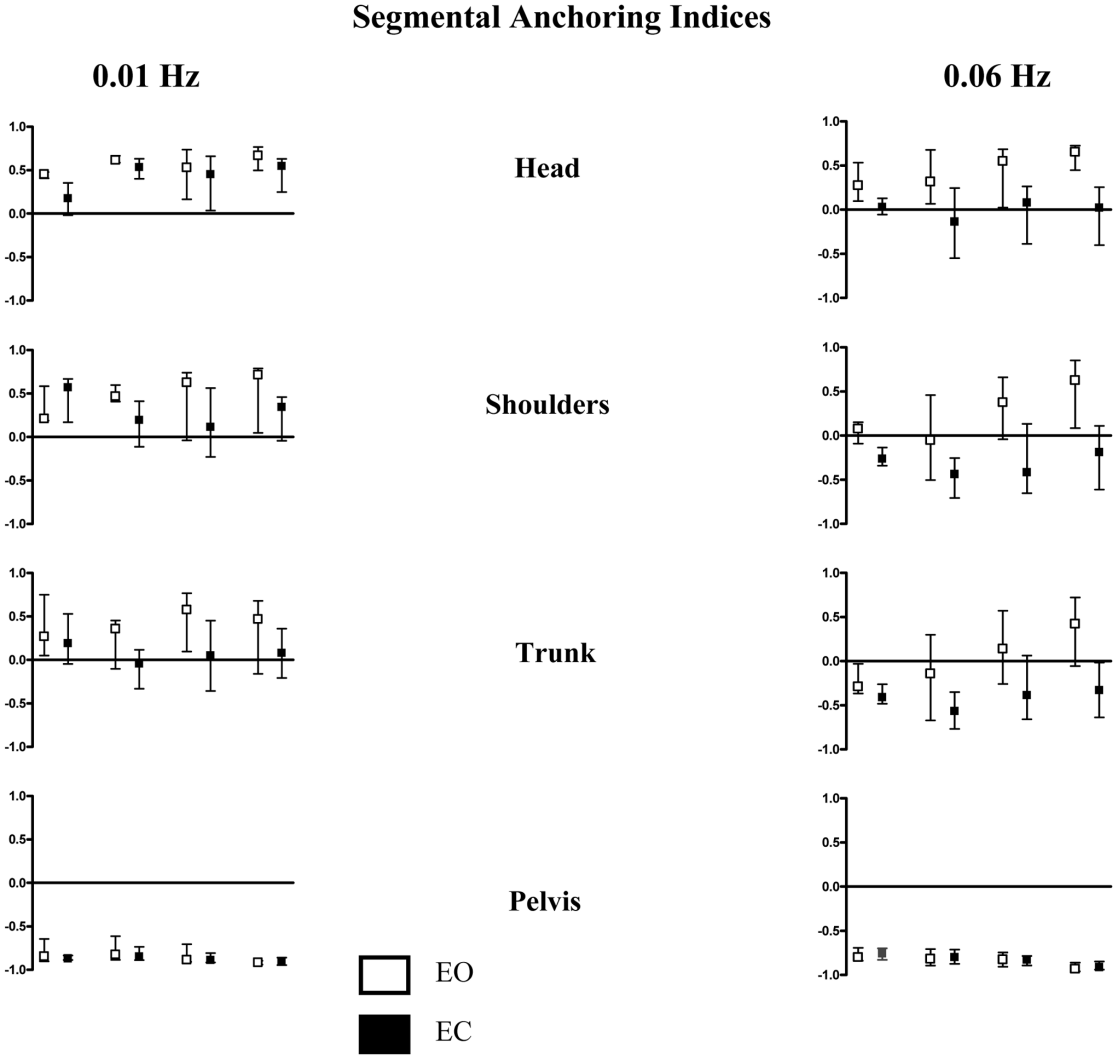
Median and quartiles of roll head, shoulders, trunk and pelvis (top to down) anchoring index, with eyes open (white) and eyes closed (black) in subjects from 5 to 15 years at 0.01 Hz (left panel) and at 0.06 Hz (right panel).

The mean anchoring indices of each segment considered with and without vision, at the lower frequency 0.01 Hz (left part) and at the higher frequency 0.06 Hz (right part), from 5 to 15 years of age, are shown in figure 7.

#### a. Age effect

***At the head level***, at 0.01 Hz, with vision, the Kruskall Wallis analysis revealed a significant age effect (EO, H (3, 51) = 8.18, p<.05). More precisely, the multiple comparison procedure revealed that head AI of the youngest subjects was significantly lower as compared with those of the oldest subjects (Q = 2.72, p<0.05). Without vision, the statistical analysis did not reveal any age effect. At 0.06 Hz, the head AI values were similar in all groups of subjects, as attested by the absence of significant statistical analysis with vision as well without vision.

***At the shoulders level***, at 0.01 Hz, with vision as well as without vision, the Kruskall Wallis analysis revealed no significant age effect. At 0.06 Hz, with vision, the Kruskall Wallis analysis revealed a significant age effect (H (3, 51) = 11.03, p<.05) with an increase of the shoulders AI from 5 to 15 years old. More precisely, the multiple comparison the multiple comparison procedure revealed that head AI of the 2 youngest groups of subjects was significantly lower as compared with those of the oldest subjects (14–15 yo) (Q = 2.77, p<0.05 and Q = 2.73 for 5–6 yo and 7–10 yo respectively).

Moreover, it is interesting to underline, that the shoulders AI were non significantly different from zero for the both youngest groups indicating the absence of a preferential segmental strategy of shoulders' stabilization whereas, for the oldest groups of subjects the AI values were positive indicating the presence of a shoulders stabilization on space strategy.

Without vision, we did not found an age effect. For all groups of subjects the shoulders AI values were negative, indicating that all subjects, whatever their aged, adopted a shoulders stabilization on the support strategy.

***At the trunk level***, at 0.01 Hz, with vision as well as without vision, the Kruskall Wallis analysis revealed no significant age effect. The trunk AI values were positive whatever the subjects' group with vision and negative without vision. At 0.06 Hz, with vision, the Kruskall Wallis analysis revealed a significant age effect (H (3, 51) = 11.56, p<.05) with an increase of the trunk AI from 5 to 15 years old. More precisely, the multiple comparison procedure revealed that trunk AI of the 2 groups of youngest subjects was significantly lower as compared with those of the oldest subjects (14–15 yo) (Q = 2.89, p<0.05 and Q = 2.67 for 5–6 yo and 7–10 yo respectively).

Moreover, it is interesting to underline, that the trunk AI were non significantly different from zero for the youngest groups indicating the absence of a preferential segmental strategy of trunk's stabilization whereas, for the oldest groups of subjects the AI values were positive indicating the presence of a trunk stabilization on space strategy.

Without vision, we did not found an age effect. For all groups of subjects the trunk AI values were negative, indicating that all subjects, whatever their age, adopted a trunk stabilization on the support strategy.

***At the pelvis level***, no significant effect of age was found concerning the pelvis AI values. Whatever the perturbation's frequency and the visual condition, for the 4 groups of subjects the pelvis AI values were negative, indicating that all subjects, whatever their age, adopted a pelvis stabilization on the support strategy.

#### b. Visual effect

In order to assess the effects of vision on each group of subjects, the segmental anchoring index values calculated with vision were compared with those measured without vision using a Wilcoxon analysis. The statistical values of this analysis are given on table 4 for 0.01 Hz and 0.06 Hz.

**Table 4:** Results of the Wilcoxon analysis testing the visual effect in anchoring index.

		EO				EC			
		5–6	7–10	11–13	14–15	5–6	7–10	11–13	14–15
**Head**	W	NS	NS	NS	−178	39	54	76	122
	p	P>0,05	P>0,05	P>0,05	P<0,001	P<0,05	P<0,05	P<0,001	P<0,05
**Trunk**	W	55	NS	NS	NS	53	62	46	NS
	p	P>0,05	P>0,05	P>0,05	P>0,05	P<0,001	P<0,01	P<0,05	P>0,05
**Shoulders**	W	55	57	NS	NS	55	79	66	182
	p	P<0,05	P<0,05	P>0,05	P>0,05	P<0,01	P<0,01	P<0,01	P<0,001
**Pelvis**	W	NS	NS	NS	NS	NS	NS	NS	NS
	p	P>0,05	P>0,05	P>0,05	P>0,05	P>0,05	P>0,05	P>0,05	P>0,05

NS: Non significant.

The statistical analysis revealed significant decreases in AI with EC as compared to EO as follows: at 0.01 Hz, at the head, shoulder and trunk level for the 7–15 yo, at the head levels for the 5–6 yo; at 0.06 Hz at the head, shoulders and trunk level for the 7–15 yo, at the head and shoulders levels for the 5–6 yo. No significant difference was found at the pelvis for any group.

We can noted that at 0.06 Hz the head AI were always positive with vision and negative without vision, indicating that all the subjects, whatever their age, loose the strategy of head stabilization on space in absence of vision.

#### c. Frequency effect

In order to assess the effects of frequency on each group of subjects, the segmental anchoring index values calculated at 0.01 Hz were compared with those measured at 0.06 Hz using a Wilcoxon analysis.The statistical values of this analysis are given on table 5 for EO and EC conditions.

**Table 5:** Results of the Wilcoxon analysis testing the frequency effect in anchoring index.

		EO				EC			
		5–6	7–10	11–13	14–15	5–6	7–10	11–13	14–15
**Head**	W	−39	−58	−64	NS	−51	−64	−66	−204
	p	P<0,05	P<0,01	P<0,05	P>0,05	P<0,01	P<0,01	P<0,001	P<0,001
**Trunk**	W	−51	−64	−56	−174	−53	−60	NS	NS
	p	P<0,01	P<0,01	P<0,05	P<0,001	P<0,01	P<0,01	P>0,05	P>0,05
**Shoulders**	W	−55	−78	−70	−198	−53	−70	−64	NS
	p	P<0,01	P<0,001	P<0,01	P<0,001	P<0,01	P<0,01	P<0,01	P>0,05
**Pelvis**	W	NS	P>0,05	NS	P>0,05	NS	P>0,05	NS	P>0,05
	p	NS	P>0,05	NS	P>0,05	NS	P>0,05	NS	P>0,05

NS: Non significant.

The statistical analysis revealed significant decreases in AI at 0.06 Hz as compared to 0.01 Hz as follows: with vision, at the head, shoulder and trunk level for the 5–13 yo, at the shoulders and trunk levels for the 14–15 yo; without vision at the head, shoulders and trunk level for the 5–10 yo, at the head and shoulders levels for the 11–13 yo, and at the head level for the 14–15 yo. No significant difference was found at the pelvis for any group.

We can underline that at the head and the shoulders levels, the AI values were positive at the lowest frequency and near from zero at the highest frequency, translating a loss of head stabilization on space strategy with the higher frequency.

## Discussion

### Prevalence of visual contribution to postural control in children and adolescents

In our study, the use of visual cues improved the subjects' postural performances in terms of orientation and stabilization of the upper body segments, at both oscillation frequencies tested. Indeed, the anatomical damping and the segmental stabilizations improved in subjects from 5 to 15 years when visual cues were available. Without vision the independent control of each segment disappeared, particularly at the higher frequency. Similar effects were highlighted in several developmental studies [3], [38], [39] showing that children' and adolescents' postural performances decreased in the absence of vision. Ferber- Viart and colleagues [15] concluded that in balance control, somatosensory inputs are primary in adults while vision predominates in children.

Probably that in response to considerable body changes occurring during childhood and adolescence, visual cues constitute the first sensory reference frame not affected by musculoskeletal growth, for efficiently organizing postural control [9]. Moreover, perceptual studies also reported the prevalence of vision in the vertical perception. Probably also that children's and adolescents' dependence on visual cues may be part of the visual typology specific to these age-groups. Studies on boys and girls, 4 to 17 years of age, using the Rod and Frame Test (RFT) have shown that visual dependence decreases with age [40]–[41]. This decrease does not show a linear pattern and the authors of these studies reported that a peak in visual dependence occurs at the age of about 6 years, 8 years and 15 years. On the basis of these visual perceptual studies, we may speculate that our groups of subjects from 5 to 15 years were presumably still dependent on visual cues. Moreover, studies performed on adults [42], [43] have shown the existence of correlations between the subjects' perceptual and postural strategies. Visual dependent subjects were found to make greater use of visual information to control their postural orientation and stabilize their body segments, which also seems to be the case in our children and adolescents.

### Somatosensory cues integration: a slow linear improvement during childhood and adolescence

It emerges from this study that children and adolescents showed negative anchoring index values at the pelvis level. These data suggest that no attenuation of the oscillatory pattern applied at the foot level by the platform was observed at the level of the pelvis in all subjects from 5 to 15 years, who used the foot support as their reference frame. In addition, the anchoring index values near zero at the shoulders and trunk level suggest that all subjects did not have a preferential strategy to stabilize the upper segments. These developmental data clearly contrast from those previously reported in adults in a study using similar conditions [30]. Indeed, adults attenuate similar support pertubations by stabilizing their pelvis, as well as other body segments, with respect to space. Although children and adolescents adopted the head stabilization in space strategy in response to slow oscillations of the support, as it was reported in adults with higher anchoring index values, we can conclude that adults use more efficient segmental stabilization strategies than children and adolescents.

Concerning the body orientation, our results clearly showed a developmental effect indicating that support oscillations were more damped with increasing age at head and shoulders levels. However, this damping was less important than those of the adults assessed with the same slow oscillations protocol [30]. Since children and adolescents were not able to use the proprioceptive information available to show similar patterns of attenuation and segmental stabilization from adults, we concluded that they showed a maturational lag in comparison with adults. From these results, in line with the literature [24], [26] it can be speculated that the sensory integration of the somatosensory cues improves slowly during childhood and adolescence.

### Possible contribution of otolithic information

Previous studies carried out by Vaugoyeau and colleagues in adults [30], [31] showed that vertical position can be maintained on the basis of the proprioceptive information alone, associated with an independent control of each body segment. Although children and adolescents used less efficient segmental stabilization strategies than adults, they surprisingly adopted the head stabilization in space strategy at 0.01 Hz without vision that disappeared at 0.06 Hz without vision.

Although the function of the vestibular system of children is still lower than that of adults even when the children are aged 15 [25], [44], this head stabilization in space in subjects from 5 to 15 years underlies a possible contribution of otolithic information in the head maintenance. Indeed, the role of the otolithic system in postural control is still a matter of debate. Indeed, the absence of otolithic static information does not prevent the healthy subjects adopting a precise postural vertical in microgravity [45]. Nevertheless, a deafferented patient seated on a platform that tilted slowly with oscillatory angular movements in the frontal plane controls her head and shoulders with the otolithic system [46]. It has also been demonstrated that when proprioceptive and visual cues are unavailable, postural control appears to require intact vestibular function [47]. Others studies, in experts gymnasts, showed that the efficiency of the otolithic inputs can be improved through a specific training to compensate for the lack of somatosensory cues [48]. According to our results, it seems that children and adolescents also have this plasticity to exploit any sensory information available for postural control, more precisely when the others are missing or probably because the considerable body's changes occurring during adolescence, may lead oldest subjects to under-use the information provided by the proprioceptive pathway [8].

### Mild differences in the sensory integration used by children and adolescents

In this study, some developmental differences emerged at 0.06 Hz when all the sensory cues were available. Indeed, the youngest subjects (from 5 to 10 years) did not succeed in using segmental stabilization in space strategies while the oldest (from 11 to 15 years) efficiently adopted an independent control of shoulder and trunk in response to the support perturbations. Except for this point, youngest as oldest subjects adopted similar damping and stabilization strategies that gradually improved with age.

Thus, the youngest as well as the oldest subjects adopted the head stabilization in space strategy at the lower and the higher frequencies. This result contrasts with previous developmental studies indicating that the head stabilization in space strategy only appears around the age of 7 years during difficult balance tasks, like walking on a narrow support [1], [7] and seems to transitory disappear during adolescence [49]. Moreover, Assaiante and Amblard [11] have reported that a transient disappearance of the peripheral visual contribution to locomotor balance takes place at around the age of 7 years, which precisely corresponds to the beginning of the effective head stabilization on space strategy while walking on a narrow support [7].

Probably the specificities of the slow oscillations protocol could explain this difference. In fact, the slow oscillations of the support includes a maximum tilt of 10° that does not represent a major balance difficulty as the walk on a narrow support can be. Thus, the head stabilization in space strategy may be task-dependent [1], [8], [50]. Moreover, the head stabilization in space strategy mainly requires the contribution of vestibular cues [1]. In fact, the participation of the vestibular system to the postural control would be major in the most dynamic situations [48]. Thus, we can conclude that at 0.06 Hz, dynamic vestibular information would not be determining for postural control. Taking into account that head stabilization in space strategy disappeared without vision, we can reasonably speculate that in this condition, the head stabilization in space strategy is mainly based on visual cues. This result emphasizes, once again, the prevalence of visual contribution to postural control in children and adolescents.

### Linear versus non linear development of postural control

Many studies in the literature reported a non-linear rate of improvement of static balance control characterized by changes in the postural control strategy occurring around 7–8 years of age [1], [28], [51]. Surprisingly, our study did not report a change in the strategy of control between these ages. Moreover, by contrast with our working hypothesis, 14–15 years of age range did not seem to constitute a specific phase in the development of sensory integration in quasi-static postural tasks as assessed with the slow oscillations protocol. In fact, we observed a linear improvement with age from 5 to 15 years concerning orientation control as well as segmental stabilization. A possible explanation is that the support oscillations constitute first an intermediate condition between static and dynamic control and second an external disturbance imposed to the subject whereas the static postural or locomotor tasks, previously reported, are based on voluntary actions.

***In conclusion***, our developmental study provided evidence that there are mild differences in the quality of sensory integration relative to postural control in children and adolescents. The results reported here confirmed the predominance of visual cues and the gradual mastery of proprioceptive integration in postural control during a large period of ontogenesis including childhood and adolescence. Youngest as well as oldest subjects adopted similar damping and segmental stabilization strategies that gradually improved with age. Lastly, sensory reweighting for postural strategies as assessed by very slow support oscillations present a linear development without any turning point between childhood and adolescence.
